# *Fusarium*-Produced Mycotoxins in Plant-Pathogen Interactions

**DOI:** 10.3390/toxins11110664

**Published:** 2019-11-14

**Authors:** Lakshmipriya Perincherry, Justyna Lalak-Kańczugowska, Łukasz Stępień

**Affiliations:** Plant-Pathogen Interaction Team, Department of Pathogen Genetics and Plant Resistance, Institute of Plant Genetics, Polish Academy of Sciences, Strzeszyńska 34, 60-479 Poznań, Poland; lper@igr.poznan.pl (L.P.); jlal@igr.poznan.pl (J.L.-K.)

**Keywords:** fungal pathogens, *Fusarium*, pathogenicity, secondary metabolites

## Abstract

Pathogens belonging to the *Fusarium* genus are causal agents of the most significant crop diseases worldwide. Virtually all *Fusarium* species synthesize toxic secondary metabolites, known as mycotoxins; however, the roles of mycotoxins are not yet fully understood. To understand how a fungal partner alters its lifestyle to assimilate with the plant host remains a challenge. The review presented the mechanisms of mycotoxin biosynthesis in the *Fusarium* genus under various environmental conditions, such as pH, temperature, moisture content, and nitrogen source. It also concentrated on plant metabolic pathways and cytogenetic changes that are influenced as a consequence of mycotoxin confrontations. Moreover, we looked through special secondary metabolite production and mycotoxins specific for some significant fungal pathogens-plant host models. Plant strategies of avoiding the *Fusarium* mycotoxins were also discussed. Finally, we outlined the studies on the potential of plant secondary metabolites in defense reaction to *Fusarium* infection.

## 1. Introduction

Mycotoxins are toxic chemicals produced by fungal species, like *Fusarium, Alternaria, Aspergillus*, and *Penicillium*, that are either phytotoxic or are harmful to human and animal health. The management of the *Fusarium* phytopathogens has been proven to be difficult due to their high genetic variability and broad host specificity [1]. The species can adapt to a wide range of habitats, including the tropical and temperate areas, and are considered as one among the most devastating plant pathogens. As hemibiotrophic pathogens, *Fusarium* colonizes the host as biotrophic fungi, and during the necrotrophic stage, they produce toxins and cellulolytic enzymes that are aimed to hijack host secondary metabolic pathways for a better establishment and uptake of host nutrients. Mycotoxin-producing *Fusarium* species are major pathogens in cereals like wheat, oats, barley, and maize [2]. Also, they can cause up to 50% yield loss in tropical fruit crops like banana and pineapple, lentils, tomato, and pea [3,4,5,6]. Fungal secondary metabolites, such as polyketides (e.g., aflatoxins and fumonisins), terpenes (e.g., T-2 toxin, deoxynivalenol—DON), indole terpenes (e.g., paxilline and lolitrems), non-ribosomal peptides (e.g., sirodesmin, enniatins, and beauvericins), alkaloids (peramine), and siderophores (ferricrocin) often play a role in triggering infection symptoms in plants [7].

Toxins fall under the category of secondary metabolites and are often over-produced as a result of external stress. The most important stimuli for mycotoxin biosynthesis are oxidative stress, nutritional stress, and light stress, other environmental factors, such as pH, temperature, water activity, fungicides, and plant-derived secondary metabolites [8]. It was hypothesized that toxin production was aimed to reduce the excess of reactive oxygen species [9]. Later, it was demonstrated that the fungal cell utilized toxins to keep the oxidative burst under control with added ecological favors [10]. Mycotoxins, such as DON, also have some functions during the infection. *Tri5* is the gene, which encodes a trichodiene synthase that converts farnesyl diphosphate to trichodiene. It was shown that *Tri5* disrupted mutants displayed a significant reduction in infection of wheat due to their inability to produce DON [11]. Moreover, it was elucidated that mycotoxins, including DON and zearalenone (ZEN), might be synthesized to weaken the other competing microorganisms during the saprophytic growth phase. The latter, which is a non-steroidal mycotoxin, has estrogenic activity and also acts as a hormone that regulates sexual reproduction in *Fusarium* [12].

Mycotoxins can be accumulated in the tissues of cereals and vegetables and become life-threatening or severely impair human and animal biological systems. Many toxins like fumonisins and trichothecenes are heat-stable and cannot be deactivated by cooking. The only way to surpass this situation is by preventing or inhibiting the production of mycotoxins in the field. So, it is necessary to understand the molecular mechanisms of *Fusarium* mycotoxins’ action during the infection process. This review explored the mechanisms of mycotoxin synthesis in *Fusarium* species under various environmental conditions along with discussing the plant metabolic pathways and cytogenetic changes that are influenced as a consequence of mycotoxin confrontation.

## 2. *Fusarium* Mycotoxins

The factors that induce mycotoxin production are still under research. However, it is understood that some secondary metabolites, such as polyols, microsporines, and pigments, are produced as a part of fungal adaptation to stressing environments [13]. Among the *Fusarium* mycotoxins, the primarily concerned ones are the trichothecenes, fumonisins, and zearalenone [14]. Understanding the biosynthetic pathways of these mycotoxins is very important and can have a great impact on their management strategies.

### 2.1. Trichothecenes

Species like *Fusarium graminearum* and *Fusarium culmorum* are particularly devastating pathogens on small-grain cereals like durum wheat, oats, rye, barley, and triticale and also are the main cause of type B trichothecene contamination in cereals that are widespread all around the world’s cereal growing areas [15]. Trichothecenes are the mycotoxins characterized at best because of their toxicity to plants and animals. They are produced after seven to ten enzymatic modifications from the primary metabolite—farnesyl diphosphate. The *TRI* cluster of genes is responsible for their biosynthesis, and each enzyme involved in the biosynthetic process has been linked correspondingly to a specific trichothecene biosynthetic gene (*TRI*) present within or outside of the *TRI* cluster which, additionally, varies in structure from species to species [16]. The cluster has also some additional genes *TRI6* and *TRI10* that encode regulatory proteins, the *TRI12* transporter, and proteins of unknown functions [17,18]. Trichothecenes block protein synthesis in eukaryotes by three distinct mechanisms: (i) interfering with the peptidyl transferase from binding the 60S ribosomal subunit, (ii) inhibiting polypeptide chain elongation, and (iii) inhibiting chain termination [19,20]. Two types of trichothecenes were classified based on the polysomal breakdown. The type I (T type) inhibits the initiation of protein translation, and type II inhibits elongation and termination (ET type) [21]. The most important type I trichothecenes are T-2 toxin, nivalenol (NIV), fusarenon X, and verrucarin A, and type II includes DON, crotocin, and verrucarol [22].

**Figure 1:**
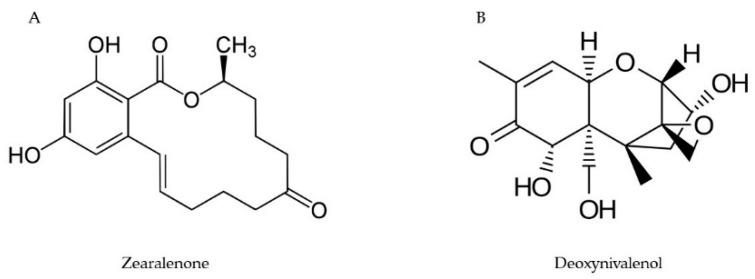
Chemical structures of zearalenone (**A**) and deoxynivalenol (**B**).

The toxicity of trichothecenes depends upon the species of the host plant. An example is a T-2 toxin and 15-acetyldeoxynivalenol, which are highly toxic to wheat and *Arabidopsis*, and 4,15-diacetoxyscirpenol is highly toxic to *Arabidopsis*, whereas neither DON nor 3-acetyldeoxynivalenol (3-ADON) are. In contrast, DON (Figure 1) and 3-ADON are more toxic to wheat than 4,15-diacetoxyscirpenol (15-DAS) [23,24]. DON is a water-soluble compound that can be easily transported via the phloem to the kernels and spikes of cereals. It also reduces germination, root, and shoot growth in wheat [25]. Similarly, T-2 and HT-2 toxins are considered to be more toxic to animals [26]. Masuda et al. [27] reported that *Arabidopsis* plants treated with T-2 toxin exhibited dwarfism and malformed morphological characteristics like shortened petiole, curled dark-green leaves, and reduced size of the cells. Consequently, it is almost impossible to predict how a particular toxin will be combated by different plant species.

### 2.2. Fumonisins

**Figure 2:**
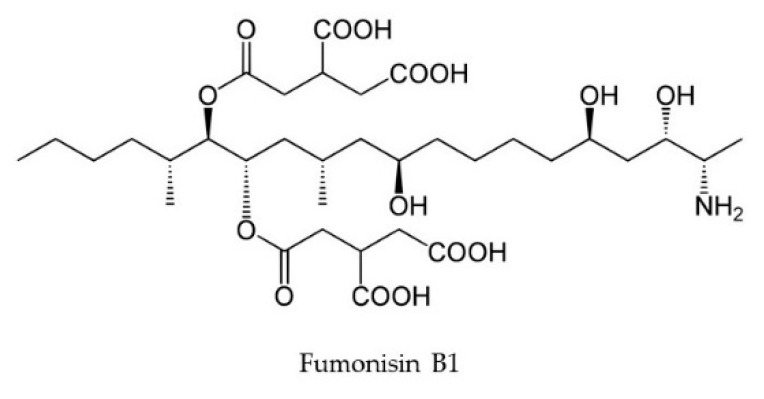
Chemical structure of fumonisin B_1_.

Fumonisins are a group of mycotoxins derived from polyketides, produced by *Fusarium verticillioides*, *Fusarium proliferatum, Fusarium sacchari, Fusarium subglutinans, Fusarium fujikuroi*, and several other species, in which the biosynthetic pathway is regulated by the *FUM* gene cluster [28,29,30,31]. This toxin group is accredited for causing equine leukoencephalomalacia, porcine pulmonary edema, and human esophageal cancer [32]. Fumonisins are also phytotoxic, and fumonisin B_1_ (Figure 2) causes damage to a wide range of cereals and other important crops. The cereals seeds contaminated with FB_1_-producing species show endosperm degradation along with the absence of protein matrix around the starch granules, possibly caused by the activity of alkaline proteases released by the fungi [33]. The toxin disrupts plasma membrane in both plant and animal species, likely to be caused by the accumulation of toxic sphingolipid intermediates [34]. These intermediates disrupt de-novo sphingolipid biosynthesis, inhibit the enzyme ceramide synthase and, hence, disrupt cell signaling and functions, alter apoptosis and replication, and are also a possible carcinogen to human beings [35]. In maize, the fumonisin-soaked seeds have shown about a 75% reduction in radicle elongation upon comparison with the control ones [36].

### 2.3. Zearalenone

Zearalenone (ZEN), formerly known as F-2 toxin, is commonly found in cereals like barley, sorghum, oats, wheat, millet, and rice (Figure 1). Because of its estrogenic activity, it causes vulvovaginitis in swine [37]. In red beet and maize, ZEN stimulates electrolyte leakage (like β-cyanin and amino acids), blocks H+ extrusion causing acidification, and, thereby, reduces the length of the root system. The toxin also reduces the activity of the ATPase enzyme in maize coleoptiles. The results of Vianello and Marci [38] suggested that ZEN could alter the permeability of plasmalemma and tonoplast.

### 2.4. Fusarins

**Figure 3:**
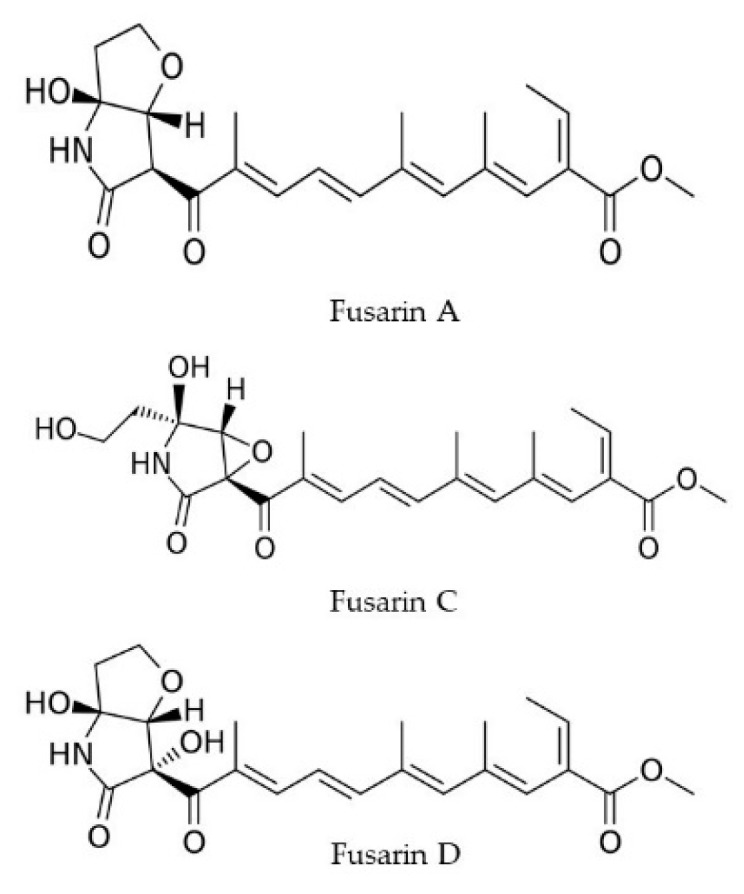
Chemical structures of fusarins.

Fusarins are polyketide compounds with a substituted 2-pyrrolidone on a polyenic chromophore [39]. They are commonly produced by *Fusarium avenaceum, F. culmorum, F. fujikuroi, F. graminearum, Fusarium oxysporum, Fusarium poae, Fusarium sporotrichioides, Fusarium venenatum*, and also by *Metarhizium anisopliae* [40]. Certain types of fusarins, like fusarin A, B, C, and D (Figure 3), were discovered in 1981 by Wiebe and Bjeldanes in Berkeley (California, USA) [41]. Among the different types, fusarin C was isolated and partially characterized, and it was also identified as a mutagen that can reverse auxotrophic *Salmonella* strains to prototrophic in the Ames *Salmonella typhimurium* test [42]. The mutagenicity of fusarin C is accredited to the presence of the C13-14 epoxide ring, whereas fusarin A and D lack this ring and are not mutagenic [43]. They are also thought to have an important role in human esophageal cancer [44].

### 2.5. Fusaric Acid

**Figure 4:**
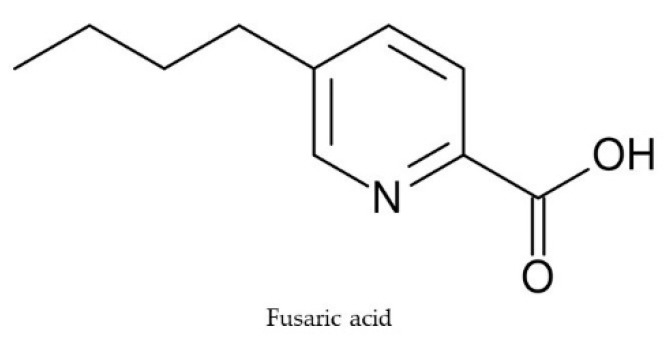
Chemical structure of fusaric acid.

Apart from fusarins, fusaric acid (FA) (Figure 4) is well-known for its phytotoxicity and is one of the first reported phytotoxins in tomato wilt symptoms caused by *F. oxysporum* f. sp. *lycopersici.* Although the toxin does not play any role in the initial infection stage, it significantly contributes to the pathogenesis process during the next stages [45]. Recently, the ion chelating activity of FA has been reported in infected tomato [46]. The strong phytotoxicity exhibited by FA is reduced by the exogenous supply of copper, iron, and zinc. Also, the toxin can increase reactive oxygen species (ROS) level, reduce the activity of antioxidant enzymes like catalase and ascorbate peroxidase, and induce cell death in detached tomato leaves [47]. External application of FA reduces the level of chlorophyll pigments and increases the total proteolytic enzyme level in tomato, eventually reducing the photosynthetic rate, cellular metabolism, and causing cell structure disruption, leading to wilt [48].

### 2.6. Moniliformin

**Figure 5:**
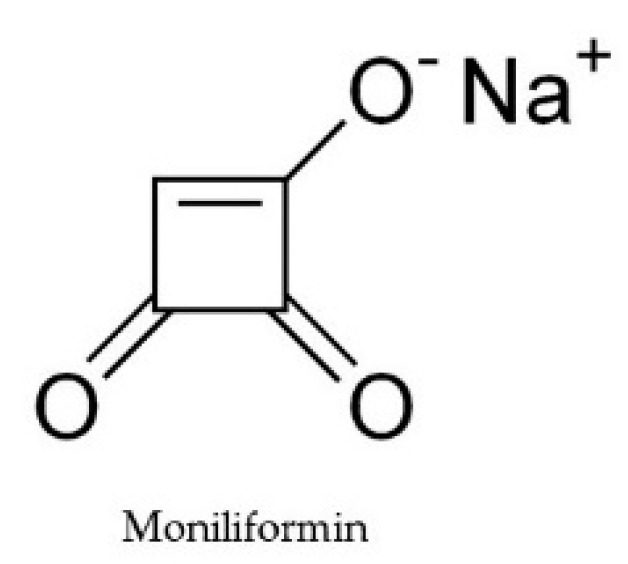
Chemical structure of moniliformin.

Moniliformin is another major contaminant in cereals produced mainly by *F. avenaceum, F. proliferatum, F. subglutinans, F. oxysporum, Fusarium chlamydosporum*, and *Fusarium anthophilum* [49]. Moniliformin (Figure 5) is less toxic than T-2 toxin, fumonisins, butenolide, and dihydrofusaric acid and has the potential to inhibit plant growth by reducing the efficacy of photosynthetic pigments [50]. The toxin can inhibit leaf development and also reduce the mass in wheat seedlings [25].

### 2.7. Enniatins and Beauvericins

**Figure 6:**
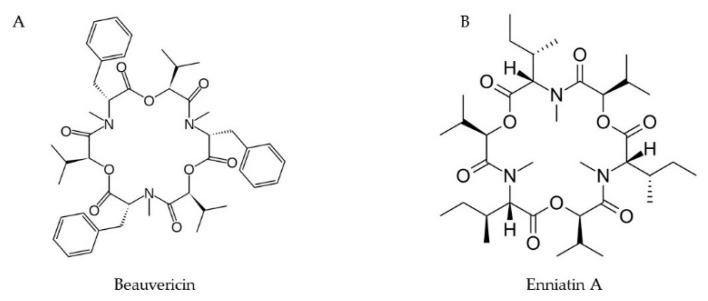
Chemical structures of beauvericin (**A**) and enniatins A (**B**).

Enniatins (ENN) and beauvericins (BEA) are cyclic depsipeptides that are produced by many *Fusarium* species and are prevalent in various food and feed items [51] (Figure 6). Data from the in vitro studies are only available regarding their toxicity and effect in the gastrointestinal tract, immunity, and steroidogenesis. Beauvericins induces DNA fragmentation and apoptosis by disrupting the mitochondrial pathways [52]. A possible role of BEA and T-2 toxin in the phytotoxicity in tomato protoplasts were reported by Paciolla et al. [53]. It was found that BEA reduced the level of ascorbic acid in the cell and collapsed the ascorbate system, leading to protoplast death.

## 3. Effect of Climate Change/Environment Factors on Mycotoxin Biosynthesis—Overview

### 3.1. Temperature and Moisture Content

The life cycle of *Fusarium* species starts with the colonization of the host, and the successful survival leads to sporulation where the spores are dispersed via wind or by rain splashes. So, it is obvious that their life cycle largely depends upon environmental factors. Variations in the environmental conditions can lead to two types of stresses in the pathogen: abiotic and biotic. Both types can impair normal metabolic functions and are followed by either adaptation, survival, or death of the fungus. It is essential to understand the factors that escort the common production of mycotoxins so that we can determine the management strategies to reduce toxin content. Reports suggest that factors, such as temperature, water activity, and growth time, has a direct influence on DON production in *F. culmorum, F. graminearum*, and *Fusarium meridionale* [54,55,56]. Llorens et al. [54] reported that the most favorable temperature for the growth of *F. graminearum* and *F. culmorum* ranged from 20 °C to 32 °C, and fungal growth was reduced at 15 °C. The expression studies of the *TRI* cluster showed that it required an optimal water activity for the expression of all the genes [57]. The optimal temperature for the production of DON is 28 °C, whereas NIV production depends on the species, strain, and also on the temperature [54].

The toxin levels are substantially higher in the Mediterranean and temperate regions where the pathogens receive extreme temperature, rainfall, and extended duration of drought [58,59]. The ear rot infection caused by *F. verticillioides* gets worse during the flowering stage, and the toxin accumulation is also favored during warm conditions of about 30–35 °C with less rainfall [60]. Cendoya et al. [61] assessed the effect of temperature (15, 25, and 30 °C) and water activity (a_w_ 0.995, 0.98, 0.96, 0.94, 0.92, and 0.88) on toxin production and fungal growth in three *F. proliferatum* strains from wheat grains in Argentina. Even though the change in those parameters did not affect much the fungal growth, it did affect the fumonisin production of the strains. They found that maximum fumonisin production was obtained at a_w_ of 0.99 at 15 °C for two strains and 25 °C for the third strain at the same a_w_ level.

Fanelli et al. [62] analyzed the effect of various light wavelengths on the synthesis of fumonisins by *F. proliferatum.* Incubation of the cultures in the light had a positive effect on the level of fumonisins and showed a difference of 40% when compared to that of the cultures grown in the darkness. Stimulation on fumonisin biosynthesis increased upon the incubation within the wavelengths ranging from long (627 nm) to short (470–455 nm) or from both sides of the spectrum. Also, red and blue light was found to be increasing the fumonisin biosynthesis when compared to all other light components, including darkness [62].

The production of fumonisin is directly governed by the expression of *FUM1* and several other *FUM* genes, the activity of which is controlled during co-regulated transcription [63]. Thus, varying environmental conditions can have a direct impact on the expression of biosynthetic genes. Nowadays, real-time RT (reverse transcription)-qPCR (RT-qPCR) techniques are used to analyze the effect of various eco-physiological factors on fumonisin biosynthesis. Water potential values may have a dissimilar magnitude in different plant parts. In maize, the water stress progressively increases during the maturation of kernels. The stress created by non-ionic solute potential increases the expression of the *FUM1* gene in *F. verticillioides*, and this finding also explains why there is an enhanced accumulation of fumonisins in maize kernels towards the stage of maturation [64]. The expression of the *FUM1* gene coincides with mild water stress (−0.7 and −2.8 MPa) and also during severe conditions (−0.7 and −10.0 MPa).

Accordingly, the production of DON and other trichothecenes is controlled by a cluster of *TRI* genes, namely *TRI5* (encoding trichodiene synthase), *TRI4* (multifunctional cytochrome P450 monooxygenase catalyzing the transition of trichodiene to isotrichodermin and trichothecene), *TRI6* (zinc finger protease transcription factor that positively regulates the biosynthetic pathway), *TRI10* (transcription factor regulating *TRI6*), and *TRI13* (cytochrome P450 oxygenase) [18]. Schmidt-Heydt et al. studied the effect of water activity and temperature on DON production and expression of trichothecene gene clusters in *F. graminerum* and *F. culmorum* [57]. The expression of all *TRI* genes tested increased between 20 and 25 °C, especially the activity of *TRI5* and *TRI4* responsible for the initiation of the biosynthetic pathway by trichodiene synthase and trichodiene oxygenase. The optimum water activity was 0.995 for the expression of all the genes in the *TRI* cluster. As the *TRI5* is the key member of the cluster involved in trichothecene biosynthesis, it was reported to be expressed in almost every condition studied. Whereas, some reports suggested that, since the activation of the *TRI* genes is completely dependent on the ZFP coded by *TRI6*, the disruption of *TRI6* and *TRI10* reduced the activity of all other genes and also reduced the levels of DON [11,65,66].

### 3.2. Effect of pH

The pH value is also considered as one of the key elements that determine the growth, development, and secondary metabolite synthesis in fungi [67,68,69]. The effect of pH on growth and mycotoxin level in fungal pathogen can be very evident in ripening fruits [70], where the pH varies continuously. In Persian lime (*Citrus latifolia* T.), the levels of mycotoxins, such as fumonisins and aflatoxins, were observed in acidic pH at the temperature of 20 °C [71]. Furthermore, the proteomics-based approach is currently being used to identify the differentially expressed proteins in varying pH and other culture conditions. *F. proliferatum* strains cultivated under pH = 5 and pH = 10 have shown significant differences in accumulated proteins after two-dimensional gel electrophoresis and matrix-assisted laser desorption/ionization/time-of-flight (MALDI-TOF/TOF) and liquid chromatography-electrospray ionization mass spectrometry (LC-ESI-MS/MS) analyses [72]. Under alkaline pH, the polyketide synthase, cytochrome P450, S-adenosylmethionine synthase, and O-methyl transferase were found to be up-regulated, which favored the biosynthesis of fumonisins. On the other hand, L-amino acid oxidase, isocitrate dehydrogenase, and citrate lyase that might inhibit the condensation of fumonisin backbone were reported to be up-regulated in acidic pH, resulting in decreased synthesis of fumonisins [72].

The role of pH in the mycotoxin elicitation in cereals is poorly recognized. Nevertheless, the effects of varying pH of in vitro cultures of *F. graminearum* on the growth and mycotoxin synthesis are available. In a low-buffered culture medium, the fungus creates extracellular acidification that results in the collateral expression of *TRI* genes and increased production of trichothecenes [73]. A certain component of the pH regulatory system called PacC transcription factor (TF) has been found to be elevated during alkaline pH as reported for the first time in *Aspergillus nidulans* [74]. The PacC contains three zinc-finger domains, and the primary translation product undergoes proteolytic processing and becomes active only under alkaline pH. Six *Pal* genes act as protease enzymes during signal perception and transduction in alkaline pH and eventually activate the alkaline-expressed genes while repressing the acid-expressed genes, including *PacC* [75]. Correspondingly, the orthologs of *PacC*, undergoing similar proteolytic cleavage for their activation, were identified in *F. graminearum* and *F. oxysporum* [76,77]. They have been assigned as *Pac1* TFs and are considered to have a direct impact on the regulation of trichothecene synthesis in alkaline pH [77]. In *F. verticillioides, Pac1* is activated in alkaline pH and strongly represses *FUM* genes, and, consequently, it is supposed to act as a repressor of fumonisin biosynthesis [78].

Meanwhile, the most commonly found fumonisin B_1_ was produced by the strains of *F. verticillioides* and *F. proliferatum* at the pH below 5 in a well-aerated condition [79]. There are reports suggesting that *F. proliferatum* is a post-harvest fruit pathogen that spoils the quality of the fruit mainly by the production of fusaric acid [80,81]. FA also causes phytotoxicity via reducing the cell membrane potential and overriding the plant defense systems, leading eventually to wilt [82]. The *F. proliferatum* strain isolated from infected banana fruit was able to produce large amounts of fusaric acid at the optimum pH of 7 [83]. Besides, the authors demonstrated a bacterial bioluminescent assay for evaluating biological toxicity caused by fusaric acid. The intensity of the luminescence emitted by the bacterium *Vibrio qinghaiensis* sp. nov. Q67 was inhibited by fusaric acid, and the character was found to be effective in measuring the FA produced by *F. proliferatum*.

### 3.3. Effect of Nitrogen Sources and Plant Extracts

The effect of various medium substrates on the ochratoxin A production by the strains of *Aspergillus ochraceus* and *Penicillium verrucosum* was studied by Skrinjar and Dimic [84]. Also, in other reports, the nitrogen source played an important role in toxin production [85,86]. The culture conditions with limited nitrogen availability increase the production of fumonisins, and, since this synthesis is regulated through a nitrogen metabolite regression mechanism, the risks posed by fumonisin contamination in food can be reduced by targeting these regulatory elements. The increased concentration of nitrogen apparently reduces the fumonisin production in vitro, and also the addition of ammonium phosphate to cracked maize grain reduces the level of fumonisin by 97% [85].

The strains of *Fusarium* isolated from different crops show increased expression of mycotoxin biosynthetic genes when grown on media supplemented with their respective host extracts. In particular, the impact of various plant extracts as oxidant stressors that elevate the levels of fungal toxins was evaluated recently. Studies showed that extracts of garlic, pea, asparagus, and maize could subsequently reduce the amount of fumonisin produced by *Fusarium* [87], while the extracts of pineapple increased the fumonisin production. Interestingly, the pea extract significantly reduced the toxin content.

The influence of essential oils made from clove, cinnamon, palmarosa, oregano, and lemongrass on the biosynthesis of ZEN and DON was analyzed by Marín et al. [88]. The effectiveness of toxin reduction presented by essential oils from cinnamon, lemongrass, and palmarosa required high humidity and optimum temperature of 20 °C and were mostly attributed to environmental factors like water activity and temperature.

Polyamines, such as putrescine, spermidine, and spermine, regulate the metabolism of nitrogen and carbon sources in plants, leading to the accumulation of various amino acids [89]. Although various polyamines have a substantial role in plant resistance to abiotic stress, very limited information is available regarding the biotic stress [90]. A study by Kawakami et al. [91] reported the effect of specific amines on the production of trichothecenes via acidification of the growth media. The possible role of the nutrient composition of maize kernels in fumonisin B_1_ biosynthesis by *F. verticillioides* revealed that amylopectin—one of the components of starch—induced the production of FB_1_, whereas, the strain with a disrupted α-amylase gene significantly reduced the toxin amount [92].

Derivatives of sucrose, such as α-glucosides, are regarded as key factors for the production of various trichothecenes in *Fusarium asiaticum* [91]. The wheat spikes after anthesis contain various carbon sources, such as sucrose and fructooligosaccharides, which facilitate the *F. graminearum* infection process and mycotoxin synthesis [93]. Also, the infection severity varies during the initial, early, and terminal stages. Oxidative stress encountered by the fungus during the interaction with plant host is considered as the most important factor, inducing the biosynthesis of mycotoxins via reactive oxygen species (ROS) modulation [8]. ROS can be very harmful to the organism by creating a state of oxidative stress inside the cell that leads to the damage of cellular constituents. The addition of hydrogen peroxide and diamide to the *Fusarium*-culturing media significantly induces the synthesis of DON and 15-acetyl-DON [94]. On the contrary, the addition of catalase enzyme down-regulates the expression of *TRI* genes and reduces trichothecene accumulation [95].

## 4. Effect of Mycotoxins on Plant Secondary Metabolite Production During Infection

### 4.1. Infection Process and Changes Inside the Host Cells

**Figure 7:**
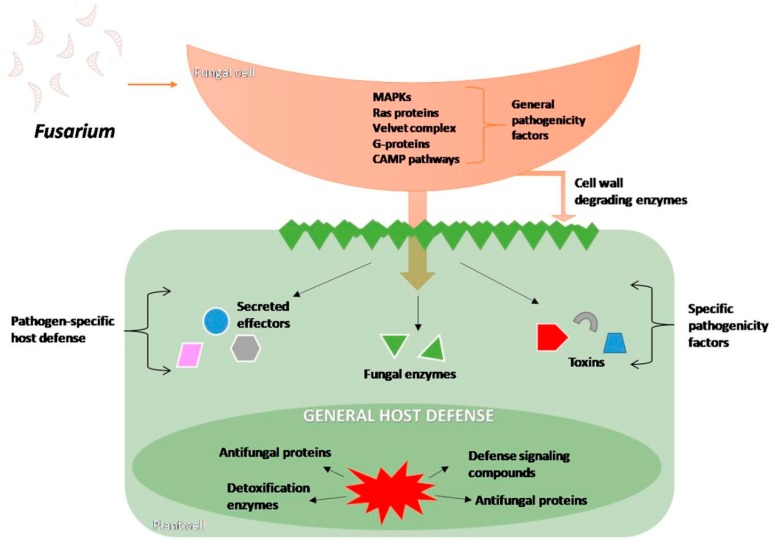
Schematic overview of the *Fusarium* pathogenicity effectors, signal transduction pathways triggered (MAPK- Mitogen-activated protein kinase, CAMP- Cyclic adenosine monophosphate), and defense reaction of host, based on [96].

The interactions between the plant and the fungus are diverse, complex, and can alter the physiology and morphology of both partners. *Fusarium* establishes the colony and destroys plant tissues by overriding the plant defense mechanisms and also by producing host-specific toxins (Figure 7).

*Fusarium* head blight (FHB) and *Fusarium* foot rot are important diseases causing severe yield loss in many crops worldwide. Depending on the infection severity, the yield loss can reach 50% for small grain cereals [97], simultaneously decreasing cereal grain quality, which in turn makes it even more vulnerable for secondary storage deterioration and mycotoxin accumulation. The infection normally requires warm and humid conditions. It is very common to identify as many as seven to seventeen species of *Fusarium* from the newly harvested cereals, but only a few of them predominate and cause diseases in the crops. This often depends on the type of host and agro-climatic conditions [97,98]. The vascular establishment of *Fusarium* species is a complex process, which involves adhesion, penetration through stomatal pores, by wounds or along the pericarp of the seeds in the case of *F. graminearum*, and subsequent colonization, both inter- and intracellular [99,100]. It is facilitated by the secondary metabolites produced by the fungus, for which the host plant initiates a defense response termed as effector-triggered immunity (ETI) or pathogen-associated molecular pattern (PAMP) triggered immunity (PTI). Penetration into the plant cell wall is achieved by the action of several cell wall degrading enzymes like cellulases, pectinases, lipases, and xylanases [101,102].

*F. culmorum* infects wheat spikes and produces the above-mentioned enzyme classes that soften and gradually dissolve the host cell wall, facilitating the invasion and absorption of nutrients [103]. Alternative studies have shown that fumonisins and AAL toxins from *Fusarium* and *Alternaria* sp., respectively, act as sphingosine analogs and inhibit ceramide synthase and sphinganine–*N*-acetyltransferase [104,105]. Since sphingolipids are the major structural component of the plant endomembrane system, inhibition of their biosynthesis leads to the malformation of the membranes and eventually to cell lysis. Primary infection initiation occurs on the peripheral surface of the plant around the infection site, and the fungal hyphae develop into the cortical area, reaching the xylem and growing upward inside the stem xylem [106]. The increased biomass during the lag phase, thus, enters a necrotrophic phase [107].

The severity of infection by *F. graminearum* and *Fusarium pseudograminearum* relays completely upon the production of the DON. Studies on an *F. graminearum* strain with mutated *TRI5* gene showed that this strain was unable to produce DON and was less aggressive during head blight disease in wheat [108]. Fungal biomass, bleaching of chlorophyll, and necrosis of florets were reduced in barley infected by the *TRI5* mutant. Normally, barley exhibits an innate resistance against trichothecenes along with the basal defense response against the pathogen. One of the host responses identified in barley during *F. graminearum* infection is the activation of genes involved in the detoxification processes that neutralize the action of trichothecenes: *UDP*-glycosyltransferases, multi-drug and toxic compound extrusion (MATE), and ATP-binding cassette (ABC) transporters, and ubiquitination of related transcripts are related to the programmed cell death, which limits the spread of the infection [108]. DON-producing strains were more aggressive, and the infection spreading from the florets to the rachis made the management even more difficult. The effect of various *Fusarium*-produced mycotoxins was studied by Packa [109] on actively dividing root tip cells of *Secale cereale*, *Triticum aestivum*, and *Vicia faba*. This cytogenetic study revealed that trichothecenes like DAS, T-2 toxin, DON, or 3Ac-DON had a profound effect on spindle fiber formation, indicating the inhibitory effect of trichothecenes on protein translation. The karyokinetic spindle disturbance failed to pull the chromosomes towards the cell poles, resulting in excessive condensation of prophase and metaphase chromosomes and subsequent accumulation of the cells in the metaphase. The mitotic index was reduced, and cell division was arrested. Especially, DON increased the cell G2/G1 ratio with an elevated 4C DNA content in the cells, indicating that the cell division was arrested in the G2 phase without entering the mitotic phase [110].

Bushnell et al. [111] demonstrated the effect of DON on the loss of chloroplast pigments. The bleaching effect of DON was greatly enhanced within 48 h after the addition of Ca^2+^ ions, indicating that the contents of chlorophyll a and b and carotenoids were reduced. The toxic effect of the DON was due to the loss of electrolytes from the cells and damaging the plasmalemma. Interestingly, DON also caused photobleaching and, although the leaves kept high amounts of electrolytes in the darkness, the cells remained green. This finding indicated that senescence was delayed at the early stages of infection and that it contributed to bleaching and cell death at a later stage.

It is assumed that DON causes also more damage to the membranes of the susceptible genotypes by increasing the efflux of K^+^ ions [111]. The activity of mycotoxins to change the properties of the plasma membrane determines their toxicity, and incorporation of these toxins into the cell membrane is detrimental to the secondary messenger systems [112,113]. Also, the electrolyte leakage value was used to determine the differential response of various genotypes to mycotoxins. This assay, however, is limited to host-selective toxins, such as victorin, HS toxin, PC toxin, and *Verticillium* toxins [114,115,116].

In defense response, the plant exhibits an increased expression of the phenylpropanoid pathway genes and synthesis of lignin, which helps to thicken the cell wall to prevent further fungal penetration. DON-producing strains were found to prevent the thickening of the host cell walls; therefore, it has been concluded that DON facilitates the spreading of infection [117]. The infiltration of trichothecenes in wheat leaves was reported to induce the production of H_2_O_2_ that steered up the cell to undergo apoptosis [118]. It was hallmarked by the occurrence of degraded DNA ladder within 24 h of infusion [119]. In tomato, proteins similar to human cell apoptotic factor PIRIN involved in the inhibition of anti-apoptotic transcription factors [120] are expected to be the reason for programmed cell death after the treatment with DON.

### 4.2. The Signaling Crosstalk for Disease Resistance

There is no cereal variety with complete resistance to the *Fusarium*-caused disease; however, there are some tolerant varieties with obvious yield decrease as a ‘cost’ of tolerating the disease. *Fusarium* head blight resistance is of five types: Type I is the resistance against initial infection; Type II against disease-spreading; Type III resistance to mycotoxin accumulation; Type IV is the kernel infection resistance; Type V for disease tolerance [121]. More than 250 quantitive trait loci (QTLs) have been identified that confer resistance, including all five groups [122]. The QTLs coming under type III resistance are involved in the detoxification pathways, which makes the plant insensitive to the toxin or resistant to the disease [123]. In maize, about 15 QTLs for *Fusarium* ear rot, as well as 17 QTLs for FB_1_ contamination, have been identified [124]. The commonly found QTLs that are highly expressed during disease resistance and detoxification pathways are stress-related protein interactor, glutathione-S- transferase, plant AT-rich sequence and zinc-binding proteins (PLATZ) transcription factor, ethylene-responsive factor-like protein-1, heat shock protein 90 (HSP), HSP 70-2, ascorbate peroxidase (APX), and the vicilin-like antimicrobial peptide 2-3.

Furthermore, some of these QTLs are associated with single nucleotide polymorphisms (SNPs) and overlapped with previously found QTLs for flowering and other disease resistance genes. Also, wheat lines carrying the QTL Fhb1 have produced glucoside derivatives of DON (DON-3-glucoside—D3G), and the D3G: DON ratio apparently increased, showing higher detoxification rates [125]. Few QTLs and tightly linked simple sequence repeats (SSR) markers have been identified in partially resistant Australian wheat cultivars [126,127]. This tight linkage between the genes makes them stable throughout generations and easier to further analyze the candidate genes.

Infiltration studies with T-2 toxin in *Fusarium*-susceptible *Arabidopsis* resulted in rapid and prolonged activation of two mitogen-associated protein kinases (MAPK), namely MPK6 and P44MAPK, which in turn activated the expression of pathogenesis related-1 (PR1) and plant defensin 1.2 (PDF1.2). Also, T-2 toxin was reported to induce an elevation of salicylic acid (SA) and jasmonic acid/ethylene (JA/ET) levels, suggesting significant crosstalk between these pathways [118]. Similarly, the *PR* genes like *PR14*, *PR5*, and also genes encoding the ABC transporters, were found to be over-expressed during the first and latent stages of infection in wheat cultivars [128].

The activation of these pathways and the severity of disease incidence relies completely on the fungal species and strains, as well as the number of mycotoxins produced. It has been shown on the *Arabidopsis* model that the host cell jasmonate signaling pathway contributes to the enhanced protection against various necrotrophic pathogens. Jasmonate-Zim-Domain proteins (JAZ) bind to various transcription factors and suppress the jasmonate-induced gene expression during an inactive state [129]. Upon pathogen encounter, a signal elicited by pathogen effector molecules induces the conversion of the chloroplastic α-linolenic acid to 12-oxo-phytodienoic acid (OPDA), which is then transported to peroxisomes, where it undergoes β-oxidation to generate JA [130]. In the cytosol, JA can be converted into active forms that mediate various cellular metabolic processes. These derivatives can then bind to the CORONATINE INSENSITIVE1 (COI1), converting it into an E3 ubiquitin ligase that ubiquitinates JAZ, thus leading to its degradation by the 26S proteasome, and to the activation of JA-induced defense pathways [129].

There are reports that species like *F. oxysporum* hijack the host oxylipin-JA signaling, mediated through the F-box protein COI1 pathway, which causes extreme wilt disease symptoms and can often result in plant death in *Arabidopsis* [131]. Zhang et al. [132] identified two JA pathway regulatory ubiquitin ligases: ring domain ligase 3 (RGLG3) and RGLG4, which regulate fumonisin B_1_-mediated apoptosis through suppressing the jasmonate pathway. The *rglg3* and *rglg4* mutant plants exhibit necrotic lesions, whereas *rglg3/rglg4* double mutants manifest less damage upon application of fumonisin B_1_, along with an increased expression of jasmonate-responsive genes like *PDF1.2*, indicating that these ligases play substantial roles in FB_1_-mediated programmed cell death.

### 4.3. Plant Strategies of Avoiding Fusarium Mycotoxins

Plants deal with mycotoxins using at least two mechanisms: chemical modification and compartmentation. Detoxification by the activity of the UDP glycosyltransferases (UGTs) can be seen prominently in *Arabidopsis* [133] and barley [108]. The microarray analysis of differentially expressed transcripts in wheat during DON treatment was reported to identify about 10 transcripts that are important for DON detoxification and are expressed during plant pathogenesis by mycotoxigenic *Fusaria* [134]. The identified transcripts included multidrug-resistant ABC transporter, two cytochrome P450 enzyme homologs (CYPs), and a uridine diphosphate-glucosyltransferase (UGT).

Similarly, the whole transcriptome analysis of trichothecene-treated barley cultivars showed the up-regulation of about 63 transcripts that have putative roles in ubiquitination, trichothecene detoxification and transportation, transcription factors, cytochrome P450s, and, finally, programmed cell death proteins [108]. F-box protein, U-box domain-containing protein, ATPases associated with diverse cellular Activities (AAA family ATPase), and nuclear transcription factor (NF) X1-type zinc finger family are the annotated proteins involved in ubiquitination. UGTs, MATE, and ABC transporters protein are encompassed for trichothecene detoxification. UGTs are involved in the transfer of glucose from UDP-glucose to the hydroxyl group at carbon 3 of the DON, as well as in the detoxification of 15-acetyl-DON. It has been reported that *Arabidopsis* possesses a superfamily of about 100 genes that encode UGTs with a consensus sequence of 42 amino acids [135]. Poppenberger et al. [133] discovered a deoxynivalenol-glucosyltransferase enzyme (AtDOGT1) that shows high similarity to the salicylic acid- and wound-inducible UGTs in other species and is expressed in the presence of DON or under stress-induced compounds like SA, ET, and JA. Similarly, several UGTs that detoxify DON were reported in *Brachypodium distachyon*, barley, and rice [136,137,138]. The functions of UGTs are not limited to detoxification; they also act on various plant secondary compounds like flavonoids, terpenes, hormones (e.g., auxin, cytokinin, salicylic acid), and many other molecules that regulate plant growth, differentiation, and disease development [139]. Biotransformation of DON to its glucoside derivatives is not only the possible conjugation strategy by plants. Warth et al. [140] reported sulfate conjugates of DON: DON-3-sulfate and DON-15-sulfate in wheat, where DON-15-sulfate was 44 times less inhibitory than DON, and DON-3-sulfate was not toxic at all.

Apart from conjugation, there are several other mechanisms embraced by the host defense machinery. Transportation of the mycotoxins out of the cells by multidrug transporters plays a potential role here and has been identified by transcriptomic studies [141]. Multidrug transporters in plants are generally categorized into four subgroups: the ATP-binding cassette (ABC) superfamily, the small multidrug resistance family (MDR), the major facilitator superfamily, and the resistance-nodulation-cell division family (RND) [142]. A fifth family called the multidrug and toxic compound extrusion (MATE) family of transporters that bind to a number of potentially cytotoxic compounds and transport them outside the cell in an ATP/proton-dependent process, has been added to these groups by Brown et al. [143]. The ABC transporters are localized in the plasma membrane, chloroplast, tonoplast, mitochondria, and peroxisomes, to carry out a plethora of functions. Transportation of the toxins outside the cells or for excretion requires its oxidation by Cyt P450s along with the conjugation by a hydrophilic compound like glucose or glucuronide [144]. The potentially toxic compound is now rendered less toxic and also prevents it from crossing the membranes via diffusion. As the final step, these conjugated toxins are carried into central vacuole or are excreted externally by ABC type proteins [145].

The MATE family of transporters expels organic compounds along with acids, hormones, and other secondary metabolites. It also takes part in heavy metal and lethal compound detoxification. In fact, a number of susceptibility genes are also contributing to the disease resistance and cross-tolerance, which is beneficial to the plants [146]. Biotransformation of potentially toxic compounds in the enzymatic reaction is very common in soil microbes of discrete taxa, and, hence, mycotoxins are not accumulated in the soil [147]. Further research in the identification and screening of this enzymatic machinery in detoxification of mycotoxins is inevitable and will be beneficial for plant protection and food safety. During biotransformation, zearalenone (ZEN) is converted into the reduced phase 1 metabolites—α-zearalenol (α-ZEL) and β-ZEL, along with its conjugated forms: glucosides, dihexosides, malonylglucosides, and pentosylhexosides [148].

### 4.4. Transgenic Plants Expressing Detoxification Genes

Genetic modification of crops either by breeding or by transgenesis constitutes the potential way to reduce *Fusarium* diseases and its mycotoxins. Tobacco plants transformed with the *PDR5* gene from *Saccharomyces cerevisiae* encoding a multidrug transporter and the *TRI101* gene from *Fusarium sporotrichioides*, which encodes a trichothecene 3-O-acetyl transferase, were reported to confer significant tolerance to 4,15-diacetoxyscirpenol (DAS) [149]. The enzyme catalyzes the 3-O-acetylation of the trichothecene ring, which is related to the self-defense for their producers [150]. A moderate amount of DAS could impede shoot and root morphogenesis and inhibit chlorophyll synthesis. The transgenic tobacco seedlings were able to produce chlorophyll as a result of a lowered amount of DAS. Likewise, transgenic rice plants expressing the *TRI101* gene demonstrated DON acetylase activity with the trichothecene pathway intermediate—isotrichodermol [151]. Although the detoxification product 3-ADON is non-toxic to tobacco or rice plants, there are several conflicting reports that it is toxic to other cereals. Transgenic wheat expressing a barley UDP HvUGT13248 was developed by Li et al. [152]. It displayed complete suppression of disease spreading in the spikes, along with the rapid conjugation of DON to D3G.

The putative active site of DON is the eukaryotic ribosomal 60S subunit protein L3 encoded by the *Rpl3* gene. Resistance to the damage caused by mycotoxins was achieved by the introduction of a site-specific mutation in the rice *Rpl3* gene that encodes the protein with a change in the amino acid at 258th residue from tryptophan to cysteine [153]. The transformed plants were able to resist the infection by the trichothecene producing strains of *F. graminearum* and were able to reduce their pathogenicity. This suggests that engineering the trichothecene target sites could help the plants to continue the production of proteins and confer better resistance against the pathogen. Another study demonstrated the relation between DON synthesis and TaFROG (*Triticum aestivum Fusarium* resistance orphan gene) and one of its interacting partner, a sucrose non-fermenting-1 (SNF1)-related protein kinase 1 catalytic subunit α (SnRK1α) [154]. TaFROG improved wheat resistance to DON, but the role of *SnRK1α* genes in disease resistance is not well known. Down-regulation of two *SnRK1α* genes, using virus-induced gene silencing, increased DON damage to the spikelets and proved the positive role of TaSnRK1αs in wheat tolerance of DON.

The toxicity of trichothecene is due to the 12C–13C epoxide ring, which can be reduced by removing the oxygen from the epoxide group to yield a carbon-carbon double bond. An epoxidase from *Eubacterium* sp. isolated from the bovine gut can enzymatically reduce different types of trichothecenes and deoxynivalenol to non-toxic de-epoxide metabolites. The enzyme converts DON into its metabolite DOM-1, the non-toxic de-epoxide of DON [155]. Although no research was done on cereals regarding the phytotoxicity of de-epoxide trichothecenes, transgenic strategies using the expression of a gene encoding de-epoxidase could be of great interest for reducing their phytotoxicity. The success rate of plant breeding and transgenesis highly depends on the quality of understanding the molecular basis of host-pathogen relationships and the probable defense responses. This can contribute to the vast arena of possibilities that can be achieved to overcome the complications caused by toxin-producing *Fusarium* species.

### 4.5. Secondary Metabolites Involved in Plant Resistance Against Fusarium

A wide range of secondary metabolites (SMs) is produced by the plant when facing mycotoxins. Apart from killing the pathogen, they either inhibit the production of the toxins or limit the spreading of the pathogen [156]. Certain SMs like phenylpropanoids and terpenoids with antioxidant potential were frequently reported as the primary line of defense against common fungal pathogens [157]. SMs with antioxidant properties, such as caffeic, sinapic, ferulic, chlorogenic, and p-coumaric acids, were reported to efficiently inhibit trichothecene B production in cereals [158]. Atanasova-Penichon et al. [156] and Picot et al. [159] reported that cell wall-bound ferulic acid, its derivatives, and chlorogenic acid were the most prominently quantified upon metabolite profiling of the *Fusarium*-infected maize kernels, along with traces of p-coumaroyl-feruloyl putrescine, diferuloyl putrescine, α-tocopherols, and carotenoids.

Biosynthesis of T-2 and HT-2 toxins by *Fusarium langsethiae* and *F. sporotrichioides* was reduced by the application of various phenolic acids to the cultures, which proves the roles of antioxidant plant metabolites as potential inhibitors of *Fusarium* toxins [160]. Ferulic acid synthesized by the host plant inhibited the transcription of *TRI5*, *TRI6*, and *TRI12* genes. Also, a sub-lethal dose of α-tocopherols blocked the production of fumonisins, whereas carotenoids had less or no effect on mycotoxin production. A comparative metabolomic study with the trichothecene-producing and non-producing (Tri^-^) strains of *F. graminearum* was reported [161] and identified the metabolites that were only produced in barley inoculated with trichothecene-producing strains: cinnamic acid, sinapyl alcohol, dihydroxylinoleic acid, geranyl chalconaringenin, dihydroquercetin, heptadecatrienoic acid, and naringin. The production of plant defense hormone (JA) was also induced after the inoculation with trichothecene-producing strain.

Benzoxazinoids are another group of secondary metabolites—the indole-derived plant metabolites produced by graminaceous plants from tryptophan in the shikimate pathway. The family of these metabolites includes about 20 compounds that are involved in the biochemical resistance against various biotic stresses [162]. In winter and spring, wheat cultivars the benzoxazinoid 2-β-glucopyranoside-2,4-dihydroxy-7-methoxy-1,4-benzoxazin-3-one (DIMBOA-glc) along with α-tocopherol, and the flavonoids homoorientin and orientin were identified to inhibit DON accumulation [163].

Generally, ROS detoxification is carried out in plants by a range of enzymes and secondary metabolites with redox potential. Enzymes like ascorbate peroxidase can directly react with ROS and can also get involved with its substrate ascorbic acid. Upon oxidation by ROS, ascorbic acid is converted into dehydroascorbate (DHA) and monodehydroascorbate (MDHA), which will be hydrolyzed spontaneously [164]. Paciolla et al. [53] demonstrated the viability reduction in tomato protoplasts co-cultured with beauvericin and T-2 toxin, as these mycotoxins induce oxidative stress in the cells by H_2_O_2_ production. The toxins were able to alter the ascorbate system in plants that are normally activated during oxidation stress. Also, there was a high degree of lipid peroxidation, DHA synthesis, and a peak in the ascorbate level in the treated cells, indicating that the plants were responding to the ROS induced by these toxins.

## 5. Conclusion and Future Perspectives

Secondary metabolites (SMs) play significant roles in virulence, development, and overall lifestyle of the fungal pathogen. In fungal-plant host interactions, they may enable pathogen actions during host infection and pathogen recognition by the plant host [165]. These interactions are responsible for very complex and puzzling relationships that are being examined using metabolomic tools [166]. Nevertheless, nearly 25% of the fungal SM gene clusters have been characterized, in contrast to the plants in which only a few have been identified [167].

Simultaneous use of genomic and transcriptomic data may allow for a greater direct understanding of genetic features of pathogenic fungi to adapt to different ecological niches and diverse pathogenic lifestyles. Mehrabi et al. [167] and Fitzpatrick [168] suggested that fungal SM biosynthetic gene clusters are an outstanding model for pathogenic lifestyle diversity [13,165]. Additionally, chromosome dynamics and/or horizontal gene transfers provide agents for pathogens to enlarge their host range [167,168]. Furthermore, the discovery of next-generation RNA-Seq technologies allows to verify the expression of SM gene clusters during different phases of infection, and manipulations of strain-unique SM genes related with host-specific virulence provide the opportunity to further explore the fungal-plant interactions [13].

A deeper knowledge of the fungus-plant interaction may give some practical benefits like understanding the nature of plant resistance to fungal diseases, which possibly could limit the loss of annual crops and might be the advantage to breeders—new plant cultivars/hybrids resilient to abiotic and biotic stresses. A closer investigation of the influence of *Fusarium* on plant biology will not only improve the scientific knowledge of the agricultural ecosystem’s functions but will also be essential for sustainable control of this important plant pathogen group.
